# Quantitative proteomic analysis of amniocytes reveals potentially dysregulated molecular networks in Down syndrome

**DOI:** 10.1186/1559-0275-10-2

**Published:** 2013-02-08

**Authors:** Chan-Kyung J Cho, Andrei P Drabovich, George S Karagiannis, Eduardo Martínez-Morillo, Shawn Dason, Apostolos Dimitromanolakis, Eleftherios P Diamandis

**Affiliations:** 1 Department of Laboratory Medicine and Pathobiology, University of Toronto, Toronto, ON, Canada; 2 Samuel Lunenfeld Research Institute, Mount Sinai Hospital, Toronto, ON, Canada; 3 Department of Clinical Biochemistry, University Health Network, Toronto, ON, Canada; 4 Department of Pathology and Laboratory Medicine, Mount Sinai Hospital, Toronto, ON, Canada

**Keywords:** Down syndrome, Trisomy 21, Amniocyte, Amniotic fluid cells, Quantitative proteomics

## Abstract

**Background:**

Down syndrome (DS), caused by an extra copy of chromosome 21, affects 1 in 750 live births and is characterized by cognitive impairment and a constellation of congenital defects. Currently, little is known about the molecular pathogenesis and no direct genotype-phenotype relationship has yet been confirmed. Since DS amniocytes are expected to have a distinct biological behaviour compared to normal amniocytes, we hypothesize that relative quantification of proteins produced from trisomy and euploid (chromosomally normal) amniocytes will reveal dysregulated molecular pathways.

**Results:**

Chromosomally normal- and Trisomy 21-amniocytes were quantitatively analyzed by using Stable Isotope Labeling of Amino acids in Cell culture and tandem mass spectrometry. A total of 4919 unique proteins were identified from the supernatant and cell lysate proteome. More specifically, 4548 unique proteins were identified from the lysate, and 91% of these proteins were quantified based on MS/MS spectra ratios of peptides containing isotope-labeled amino acids. A total of 904 proteins showed significant differential expression and were involved in 25 molecular pathways, each containing a minimum of 16 proteins. Sixty of these proteins consistently showed aberrant expression from trisomy 21 affected amniocytes, indicating their potential role in DS pathogenesis. Nine proteins were analyzed with a multiplex selected reaction monitoring assay in an independent set of Trisomy 21-amniocyte samples and two of them (SOD1 and NES) showed a consistent differential expression.

**Conclusions:**

The most extensive proteome of amniocytes and amniotic fluid has been generated and differentially expressed proteins from amniocytes with Trisomy 21 revealed molecular pathways that seem to be most significantly affected by the presence of an extra copy of chromosome 21.

## Background

Down syndrome (DS) presents with a constellation of symptoms that are attributed to complete or partial triplication of human chromosome 21. Trisomy 21 (T21) is the most common human chromosomal anomaly, affecting approximately 1 in 750 live births in North America. The range and severity of phenotypic features of DS vary from individual to individual. For example, cognitive impairment is nearly universal among the DS-affected individuals, whereas congenital heart diseases are found in approximately 40 to 50% of them. Despite the high prevalence of DS and early identification of the cause (T21), its molecular pathogenesis has been poorly understood and specific treatments have consequently been practically unavailable.

Pregnancy progression and fetal development involve complex feto-maternal physiological processes that rely on intricate interactions of multitudes of genes and proteins. Therefore, the balance among these interactions will be compromised at more than one level when a major disturbance occurs. Large-scale investigations to understand the pathophysiology of DS, thus far, have focused on the mRNA level, which aimed to compare gene expression levels between the chromosomally normal (CN) and T21 status. A number of genes that showed over- or under-expression in these studies have been proposed to be responsible for DS phenotypes: *APP, BACH1, TIAM1, SOD1, SYNJ1, OLIG1, OLIG2, IFNAR1, IFNAR2, IFNGR2, GART, ITSN1, DSCR1, CBR1, CBR3, DOPEY2, MORC3, CLDN14, SIM2, HLCS, PIGP, TTC3, DSCR3, DYRK1A, KCNJ6, ERG, Ets2, HMGN1, PCP4, DSCAM, BACE2,* and *S100β*[1-5]. A major disturbance such as an extra copy of chromosome is subsequently reflected at the level of protein production and expression, and as the end-players that constitute the functional units of genes; proteins are of great value to analyze, in order to elucidate altered molecular pathways. We therefore hypothesized that identification of proteins that are involved in altered biochemical pathways, via quantitative analysis of the amniocyte proteome, will provide insights into the causes of DS phenotypes.

Amniotic fluid can be divided into two major components: supernatant fluid and free-floating fetal cells called amniocytes (also known as amniotic fluid cells). The proteome of the supernatant fluid has been actively studied, in pursuit of biomarker discovery for various prenatal conditions, including DS [6-8]. However, the proteome of the supernatant fluid poorly reflects intracellular or molecular processes, because the intracellular proteome of fetal tissue is inadequately represented. Amniocytes are shed from all three germ layers of the fetus, and some of these cells that originate from embryonic and extra-embryonic tissues show stem cell-like properties, enabling prolonged culture [9,10]. Although amniocytes have long been used for routine prenatal diagnosis for a variety of fetal abnormalities, characterization of the types and properties of cells that exist in amniotic fluid has not yet been completed [10]. Initial classification of amniotic fluid cells was reported in the 1980s, grouping them into epithelioid, amniotic fluid-specific and fibroblastoid types, based on their morphological and growth characteristics [11]. Recently, amniocytes are recognized as a rich source for pluripotent stem cells which may be useful for therapeutic purposes. In one study, human and rodent amniotic fluid cells expressing stem cell markers were isolated, and were successfully induced with growth factors to differentiate into adipogenic, myogenic, osteogenic, neuronal, endothelial, and hepatic lineages [12].

Since amniocytes with T21 are expected to have a distinct biological behavior from CN amniocytes, we hypothesize that relative mass spectrometry-based quantification and comparison of proteins produced from trisomy and euploid amniocytes will reveal dysregulated molecular pathways. To elucidate the affected pathways and networks, we used stable isotope labeling with amino acids in cell culture (SILAC) to perform an unbiased relative quantitation of amniocyte proteins. SILAC offers global quantitation with high labelling efficiency with minimal sample manipulation and technical variations. In the second part of the present study, candidate proteins were selected based on the quantitative analysis, to represent the potentially dysregulated networks in amniocytes with T21. The final part involved verification of the candidates via developing selected reaction monitoring (SRM) assays to quantitatively assess the differential expression in individual amniocyte samples, obtained at various gestational weeks in the second trimester.

## Results

### Optimization of amniocyte culture and labelling

Our preliminary experiments showed that there were no significant morphologic differences between CN and T21 amniocyte cultures up to approximately 8 doubling times, beyond which point T21 amniocytes failed to thrive. All SILAC-labeled cells were harvested after a minimum of 5 doubling times. One confluent T-175 (17,500 mm^2^ surface area) flask contained approximately 5 × 10^6^ cells, which yielded approximately 1 mg of secreted proteins. Amniocytes were grown in serum-free media (without AmnioMax Supplement and fetal bovine serum) for 48 hours before harvest, to ensure that the harvested cells are not contaminated by exogenous proteins. The incubation period of 48 hours in the serum-free media was optimized to maximize secreted protein concentration while minimizing cell death.

### Identification and quantification of proteins by mass spectrometry

**Figure 1:**
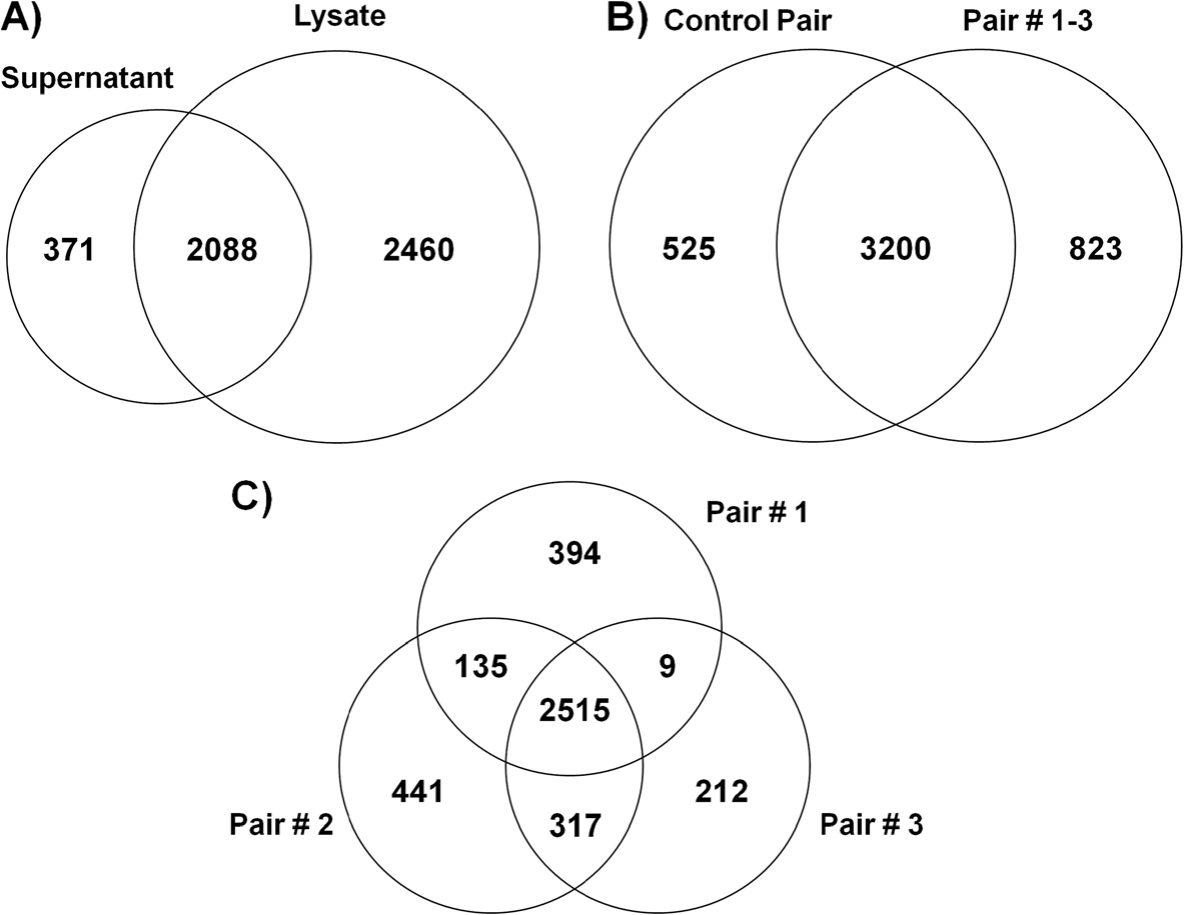
***The amniocyte *proteome. (A)** A total of 4919 unique proteins were identified from supernatant and lysate of amniotic fluid cells. **(B)***Amniocyte lysate* proteome: a total of 4548 proteins were identified from four pairs of amniocyte lysate (control pair and experimental pairs 1–3). The control pair consisted of “heavy” labeled amniocytes obtained from one euploid fetus and “light” labeled amniocytes from another euploid fetus. Each experimental pair consisted of “heavy” labeled amniocytes obtained from fetus with T21 and “light” labeled amniocytes obtained from euploid fetus. **(C)** Amniocyte lysate proteome of each individual experimental pair: a total of 4023 proteins were identified in these pairs.

To account for biovariability, we created a “control” pair, which consists of a mixture of equal amount of proteins from two separate amniocyte cultures originating from two different individuals of the same gestational age (both cytogenetically normal; CN:CN pair). A total of three “experimental” pairs were created similarly, by combining equal amounts of T21 amniocytes and CN amniocytes matched for gestational week (CN:T21 pairs). A total of 4919 unique proteins were identified from the amniotic fluid cell proteome (lysate and supernatant) at the false positive rate of 1% at both the peptide and protein level (Figure 1A). More specifically, 4548 unique proteins were identified from the lysate, and 91% of these proteins were quantified using MaxQuant (Additional file 1). From the supernatant (amniocyte conditioned media), 2459 unique proteins were identified (Additional file 2). Out of 4548 identified proteins from the lysate, 3200 of them were common between the control pair and experimental pairs 1–3 (Figure 1B). Moreover, out of 4023 proteins identified in the experimental pairs from the lysates, 2515 were found in the three pairs and 2976 in two of them (Figure 1C). Similar results were found in the supernatants. MS proteomics data have been submitted to the ProteomeXchange consortium (submission reference: 1-20130129-76233).

### Quantitative analysis to identify aberrantly expressed proteins in lysates

MaxQuant generates the ratios between “heavy”-labelled versus “light”-labelled proteins based on razor peptides, and normalizes the ratios so that the median of the logarithms of peptide ratios would be equal to zero. We thus obtained the normalized ratios and plotted proteins with statistically significant ratio values, to observe fold changes. This fold-change analysis of the lysate proteome (n = 4548) revealed that a total of 3593 proteins showed statistically significant “heavy” to “light” ratios. The mean normalized ratio was 0.91, with the vast majority of proteins showing less than two-fold increase or decrease, signifying little difference in the expression of the majority of proteins between the CN and T21 conditions.

Rather than applying an arbitrary cut-off value for fold-changes, two standard deviations from the control pair (CN:CN) was applied to the list of proteins of each experimental pair (CN:T21) to identify proteins with potentially significant differential expression. After removing the proteins that showed significant differential expression (outside of two standards of deviation) for the control pair (CN:CN), as well as reverse hits and contaminants, a total of 1135 proteins constituted the initial list of candidates. The next step was designed to maximize the number of proteins that show a true difference, with the least number of false-positives. We removed proteins that showed inconsistent fold-change between different biological replicates, based on a few razor peptides, and 904 proteins remained. The top molecular and cellular functions of these 904 proteins are represented in Additional files 3 and 4. Finally, these 904 proteins were manually checked for consistency between the ratios for different peptides of each protein, as well as for consistency in the pattern of expression of experimental pairs, and only those that show consistency with both criteria were retained. Sixty proteins, called “high probability” proteins, showed a significantly decreased (n = 29) or increased (n = 31) expression in T21 amniocytes (Tables 1 and 2).

**Table 1:** Proteins that show decreased expression in T21 amniocytes (n = 29)

**Gene name**	**Protein name**	**Ratio (H/L)**^**1**^
AKAP12	A-kinase anchor protein 12	0.41
APOA1	Apolipoprotein A-I	0.07
APOC3	Apolipoprotein C-III variant 1	0.12
ARHGEF2	Rho guanine nucleotide exchange factor 2	0.52
CNBP	cDNA FLJ77718	0.42
CTRB1	cDNA FLJ77335, highly similar to Homo sapiens chymotrypsinogen B1 (CTRB1), mRNA	0.12
ERC1	ELKS/RAB6-interacting/CAST family member 1	0.63
FBLIM1	Filamin-binding LIM protein 1	0.62
FHL3	Four and a half LIM domains protein 3	0.59
HMGA2	HMGA2e	0.52
HPX	Hemopexin	0.22
ICAM1	Intercellular adhesion molecule 1	0.62
IGF2R	Cation-independent mannose-6-phosphate receptor	0.65
LTF	Lactotransferrin	0.05
MARCKSL1	MARCKS-related protein	0.58
MCAM	Cell surface glycoprotein MUC18	0.45
NES	NES protein	0.18
NUBP1	Nucleotide-binding protein 1	0.30
PCK2	cDNA FLJ50710, highly similar to Phosphoenolpyruvate carboxykinase (GTP), mitochondrial (EC 4.1.1.32)	0.57
PGPEP1	Pyroglutamyl-peptidase 1	0.65
POSTN	Periostin	0.25
PPIF	Peptidyl-prolyl cis-trans isomerase, mitochondrial	0.20
PZP	Pregnancy zone protein	0.11
SDCBP	Syndecan binding protein (Syntenin)	0.46
SLC2A1	Solute carrier family 2, facilitated glucose transporter member 1	0.52
SOLO	Protein SOLO	0.39
TAF15	TATA-binding protein-associated factor 2N	0.39
TNS1	Tensin-1	0.57
TRIP6	Thyroid receptor-interacting protein 6	0.64

1. Heavy/light ratio with SILAC method, calculated as the average of three experimental pairs when available. Only proteins which showed decreased ratio for different peptides, as well as consistency in the pattern of expression of experimental pairs were selected.

**Table 2:** Proteins that show increased expression in T21 amniocytes (n = 31)

**Gene name**	**Protein name**	**Ratio (H/L)**^**1**^
AK6	Adenylate kinase isoenzyme 6	2.70
AMIGO2	Amphoterin-induced protein 2	8.92
ARSA	Arylsulfatase A	1.71
CD9	CD9 antigen	2.48
CNN3	Calponin-3	1.82
COL8A1	Collagen alpha-1(VIII) chain	1.93
CPA4	Carboxypeptidase A4	4.84
CRYAB	Alpha-crystallin B chain	2.70
CTSZ	Cathepsin Z	1.96
DDAH1	N(G),N(G)-dimethylarginine dimethylaminohydrolase 1	2.50
DNPEP	Aspartyl aminopeptidase	6.19
DPP7	Dipeptidyl-peptidase 2	2.31
GREM1	Gremlin-1	2.78
LCRMP	Collapsin response mediator protein 4 long variant	4.38
LPCAT2	Lysophosphatidylcholine acyltransferase 2	2.51
MFI2	Melanotransferrin	2.91
MYH10	Myosin-10	2.47
NAAA	N-acylethanolamine-hydrolyzing acid amidase	2.83
NAGLU	Alpha-N-acetylglucosaminidase	1.73
P4HA1	Prolyl 4-hydroxylase subunit alpha-1	1.98
PFKL	6-phosphofructokinase, liver type	1.98
PLOD2	Procollagen-lysine,2-oxoglutarate 5-dioxygenase 2	7.77
PPME1	Protein phosphatase methylesterase 1	1.82
PYGL	Glycogen phosphorylase, liver form	1.81
S100A10	Protein S100-A10	2.34
SFXN1	Sideroflexin-1	1.71
SIAE	Sialate O-acetylesterase	1.87
SLC25A4	ADP/ATP translocase 1	2.24
SOD1	Superoxide dismutase [Cu-Zn]	1.91
TPM2	Tropomyosin beta chain	2.70
UAP1	UDP-N-acetylhexosamine pyrophosphorylase	1.96

1. Heavy/light ratio with SILAC method, calculated as the average of three experimental pairs when available. Only proteins which showed increased ratio for different peptides, as well as consistency in the pattern of expression of experimental pairs were selected.

### Construction of networks using bioinformatic databases

**Figure 2:**
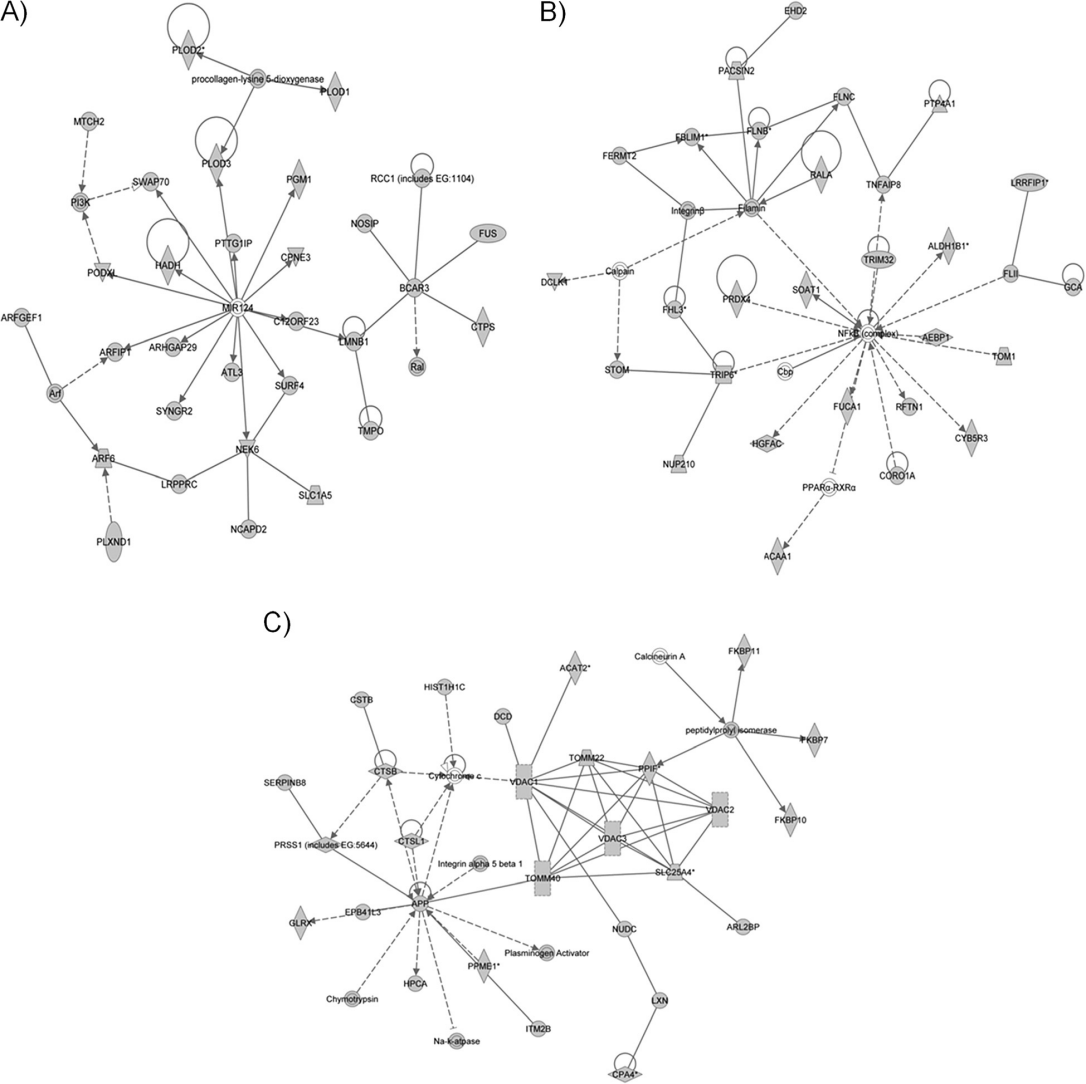
**Top three networks that are potentially disrupted in amniocytes affected with Down syndrome. (A)** A network containing 34 associated proteins, 28 of which were identified in our list of 904 proteins. This network is involved in infection mechanism, cellular assembly and organization, and cardiovascular diseases. **(B)** A network containing 35 associated proteins, 30 of which were identified in our list of 904 proteins. This network is involved in cell morphology, hematological system development and function, and humoral immune response. **(C)** A network with 35 associated proteins, 29 of which were identified in our list of 904 proteins. This network is involved in cellular assembly and organization, lipid metabolism, and organismal development.

Using the Ingenuity Pathway Analysis (IPA) software, we analyzed the list of 904 proteins to identify molecular pathways that may be directly affected due to the identified expression changes. A total of 25 pathways were identified, each containing a minimum of 16 proteins from the 904 protein list. Some of the functions and pathways include: cell morphology, hematological system development, humoral immune response, lipid metabolism, organismal development, cardiovascular disease, genetic disorder, metabolic disease, protein degradation, embryonic development, cancer, neurological disease and tissue development. The top three pathways with the highest scores (highest number of proteins that constitute the pathway being represented in the list of 904 proteins) are shown in Figure 2. Ingenuity Pathway Analysis also identified diseases and disorders, molecular and cellular functions, and physiological system development and functions for the 904 proteins. The top 5 disorders associated with these proteins were: cancer, genetic disorder, neurological disease, skeletal and muscular disorders, and cardiovascular disease. The top 5 molecular and cellular functions included: cellular movement, cell-to-cell signaling and interaction, cell death, lipid metabolism, and molecular transport. The top 5 physiological system development and functions included: tissue development, skeletal and muscular system development, cardiovascular system development and function, organismal development, and hematological system development.

### Selection and verification of candidates by SRM

From the list of “high probability” proteins (Table 1 and Table 2), candidates for further verification via multiplexed SRM assay were selected based on a number of criteria. First, proteins must be present at relatively high-abundance in amniocytes in order to be robustly and reproducibly identified by SRM assays. Second, proteins that showed greater than two-fold difference between “heavy” and “light” conditions were preferred. Third, proteins must contain unique proteotypic peptide sequences to avoid ambiguity. Finally, proteotypic peptides must meet certain requirements to facilitate selective and sensitive SRM analysis (see Methods). As a result, nine proteins were selected for multiplexed SRM assays: AKAP12, IGF2R, LCRMP, MCAM, NES, PLOD2, PYGL, SOD1 and TPM2. Ten peptides representing seven housekeeping proteins were included in the SRM assay as secondary internal standards: GAPDH, RPL27A, RPS3, TALDO1, TUBB, TUBB2C and UBB. The average H:L ratio of these housekeeping proteins from the SILAC results was 1.02 (Additional file 5). We used correlation of LC retention time between discovery and SRM gradients to confirm the identity of selected peptides, as described in more detail elsewhere [13]. More detailed peptide information, parameters of our SRM method, raw values, and coefficients of variation (CVs) can be found in Additional files 6, 7, 8, 9, 10, 11.

Two of these nine proteins, NES and SOD1, showed a highly significant differential expression (p < 0.001) in four out of five amniocyte pairs (Table 3). SOD1 expression was consistently elevated in trisomy amniocytes and NES showed marked decrease in expression.

**Table 3:** Comparison of T21/CN ratios between SILAC and SRM analysis from a total of 8 experimental pairs (3 pairs for SILAC and 5 pairs for SRM experiments)

**Gene**	**T21/CN ratios according to gestational age (weeks)**							
**Name**	**15.1**	***15.5***	**15.9**	**16.3**	***16.4***	**17.3**	**18.6**	***21.5***
NES	0.79*	*0.19*	0.34*	0.31*	*0.17*	**1.26**	0.67*	*N/A*
SOD1	1.54*	*1.98*	1.85*	1.46*	*1.74*	1.75*	**1.16**	*2.02*

T21, Trisomy 21; CN, chromosomally normal; N/A: Not available.

Bolded: Ratios (T21/CN) from SRM analysis.

Italicized: Ratios (T21/CN) from SILAC analysis.

*Statistically significant ratios at p < 0.001, according to Student’s *t*-test when the six replicates where compared.

Only the results of two proteins with highly significant fold-change in four out of five sample pairs (SRM) are displayed.

## Discussion

With the advent of mass spectrometry and bioinformatic platforms, high-throughput proteomic studies for different tissues, under various differentiation stages or disease conditions, have proliferated in the literature. Among a few quantitative proteomic techniques, SILAC has recently gained popularity for global-scale analysis of proteins in different cell conditions [14]. One notable advantage of this metabolic labelling technique is that nearly all peptides of all proteins can contribute to quantification, unlike other labelling techniques that target a group of peptides with certain characteristics to be labelled. We hence utilized SILAC to identify differences in the proteome of amniotic fluid cells from T21-affected versus CN fetuses, to identify molecular pathways that are responsible for DS pathogenesis.

The next major step after a large-scale discovery phase is selection of the most promising candidates and verification in individual samples by more elaborate quantification methods. Our initial filtering criteria for selecting candidates were based on differences between the control pair (CN:CN) and the experimental pairs (T21:CN). For example, when we considered proteins with differences exceeding 3 standard deviation in H/L ratios, the control pair showed 38 proteins, whereas the experimental pairs showed 150 to 300 proteins. These findings suggest that a large number of amniocyte proteins are expressed in different amounts between the CN and T21 conditions.

There are at least two reasons as to why our quantification based on SILAC may potentially have a relatively large variability. First, amniocytes in primary culture do not represent a homogenous population, unlike most other cell cultures. It has been observed previously, as well as in the current study, that only a subset of amniocytes survive after a few doubling times and the amniocyte cultures become relatively homogeneous, although the exact nature of these cells are yet to be determined [10]. Second, the amniocytes used in this study originated from different individuals. Therefore, the results were expected to be significantly more variable, compared to studies that use immortalized cells from one individual. Given that proteins that show differential expression in only one experimental pair may be due to analytical variability, only proteins that showed differential expression across two or more experimental pairs from our initial list of 904 proteins were retained for further analysis. Here, we employed SRM assay for verification of SILAC data, since we have previously validated its accuracy and effectiveness for verification of candidates in amniotic fluid [15].

Network modeling suggested that a number of pathways include multiple proteins that are found in our list of dysregulated proteins (Figure 2). For example, a pathway that includes NF-κB was one of our top 3 pathways, and NF-κB, along with NFATc, has been implicated in the dysregulation of DS candidate region-1 [16,17]. Another pathway that includes APP was one of our top 3 pathways, and 29 out of the 35 involved proteins of this particular network were identified in our list of 904 proteins that seem to be dysregulated. *APP* gene encodes a transmembrane protein called amyloid precursor protein in humans, which can be sequentially cleaved by the action of the β and γ secretases, to produce amyloid-beta (Aβ) peptides. APP protein and its peptides seem to contribute to the pathogenesis of DS by both gain of toxic functions and loss of normal biological functions. Aβ42 peptide is the main constituent of amyloid plaques that are a hallmark of Alzheimer’s disease, and recent studies have suggested that the cognitive decline in Alzheimer’s is mediated by reduction of synaptic plasticity attributed to the Aβ plaque formation [18]. Aβ peptides can also cause cerebral amyloid angiopathy, as these peptides aggregate to coat cerebral blood vessels. Plaques indicating amyloid angiopathy have also been observed in DS-affected brains [19]. Although the exact function of APP is unknown, APP seems to play an important role in differentiation or migration processes of neural stem cells. *In vitro* studies have shown that APP is required for differentiation of neural stem cells, and *in vivo*, it was shown that neural stem cells cannot migrate or differentiate in an APP-knockout mouse [20]. Our previous study showed that APP expression in amniotic fluid is increased by two-fold in DS-affected pregnancy, as early as the 16^th^ week of gestation [6]. Based on these previous and our current findings, we can hypothesize that APP metabolism is altered at an early stage of fetal development, and its degree of alteration may be one of the most significant, among numerous molecular pathways that are implicated in the development of DS phenotypes.

Several of the candidate proteins have also been directly or indirectly associated with various symptoms of DS in previous studies. The results obtained for SOD1 and NES seem to be particularly consistent. The *SOD1* gene is located on chromosome 21 and it encodes for superoxide dismutase, a ubiquitous protein that is involved in the clearance of free radicals produced within cells. Two types of neural pathologies are associated with this protein. First, pathogenic variants of this protein are prone to proteosomal degradation by ubiquitination processes, and such defects have been associated with amyotrophic lateral sclerosis type 1 (ALS1), a neurodegenerative disorder affecting upper and lower motor neurons [21]. Secondly, SOD1 proteins, both wild-type and variants, have a tendency to form fibrillar aggregates, and these aggregates have cytotoxic effects, resulting in neurodegeneration. Increases in SOD1 and APP were studied together, and only when combined, the double transgenic mice showed severe morphological damage [22]. Our results showed that SOD1, unlike other candidates, was consistently upregulated in T21-amniocytes compared to the controls, and this finding supports the traditional gene-dosage hypothesis even at the protein level. The hypothesis predicts increased expression of genes encoded in chromosome 21 [23], and previous studies at the mRNA level have showed mostly supportive results [24-26].

Unlike SOD1, there is little information available for NES. This protein seems to be down-regulated according to the results of the present study. NES is an intermediate filament protein that has been associated with Creutzfeldt-Jakob syndrome and pathologic neovascularization. It is expressed in various parts of the human body, including brain, eyes, ovaries, skin, and some pathologic tissues such as glioblastoma. NES expression is also strongly observed in stem cells of the central nervous system in the neural tube, and it has been speculated that it has an important role in central nervous system development [27]. Upon terminal neural differentiation, NES is downregulated and replaced by neurofilaments.

Although bioinformatic databases allow easy annotation of candidates for their function, tissue expression, and potentially involved pathways, understanding of their function must be done within the context of the cell type and state of the cells. Since amniocytes represent a relatively heterogeneous population that has not been fully characterized, speculating on each protein function in the amniotic fluid cell proteome should be approached with caution. For example, there may be an array of proteins that have been well-described in fully differentiated cells, although the same proteins may be actively involved in development and/or cellular differentiation during fetal growth. Therefore, information on their developmental functions from bioinformatic repositories may be very limited. Also, expression of proteins in terminally differentiated cells can be quite different from expression in stem cell-like cells. Moreover, gene dosage clearly depends on the biological function of the product of the gene, including enzymes, structural proteins, transcription factors, intracellular signaling molecules, cell surface markers, and receptors.

There are a few limitations of this study, which originate from the nature of the samples. For example, the heterogenous nature of amniotic fluid cells can introduce false-positives into our list of proteins that reflect DS pathogenesis, warranting a verification step. Also, the heterogeneity of the disease phenotypes and the degree of severity make the analyses more difficult. For example, 50 to 60% of DS individuals suffer from congenital cardiac defects, and some of the altered pathways for heart development could or could not be captured in our candidate list, since not all DS fetuses are affected. Even for the universal phenotypes, such as cognitive development, there is a wide range of severity; therefore “signature proteins” for any of the phenotypes could potentially be missing from our list, especially at such an early stage of development.

## Conclusions

In summary, this study identified over 4,900 proteins from primary amniocytes via proteomic discovery experiments, providing the most extensive proteome data for amniocytes, while quantifying over 85% of the identified proteins via the SILAC technique. Quantitative analysis showed that at least 900 proteins were potentially dysregulated in amniocytes with T21. The bioinformatic molecular analyses revealed multiple pathways that seem to be most significantly affected by the presence of an extra copy of chromosome 21. Further investigations of these pathways in fetal tissue may help elucidate molecular mechanisms that are directly responsible for DS features. We also designed targeted SRM assays for candidate verification and identified two proteins (SOD1 and NES) that could be involved in the molecular pathogenesis of DS during fetal development.

## Methods

### Amniotic fluid cell culture

A total of three T21 and five CN amniocyte samples were collected by amniocentesis from women at 15 to 21 weeks of gestation, undergoing prenatal diagnosis. These amniotic fluid cells were a fraction of the cells obtained for cytogenetic analysis, and they were grown to confluency in T-12.5 cm^2^ flasks for approximate 10 to 14 days in 50% (v/v) AmnioMax C100 combined media (17% AmnioMax C100 Supplement, 83% AmnioMax C100 Basal media) and 50% Chang Medium D, at the Cytogenetics Laboratory of Mount Sinai Hospital. Once chromosomal status (e.g. CN or T21) was confirmed and each flask was confluent, we harvested approximately 50% of these cells as the initial population for SILAC and placed them in new T-12.5 cm^2^ flasks. Cells from an individual constituted a single sample without pooling at any step, except for 1:1 mix (light/heavy) for SILAC analysis.

The study protocol was approved by the Institutional Review Board of Mount Sinai Hospital. Informed consent was obtained from all participants. The study was performed in accordance with the Declaration of Helsinki Principles.

### Stable Isotope Labelling by Amino acids in Cell culture (SILAC) Media Composition

SILAC media were prepared from customized Dulbeco’s Modified Eagle’s Medium (DMEM) devoid in two essential amino acids: L-arginine and L-lysine (AthenaES). Heavy amino acids, L-Arg6 (^13^C) and L-Lys8 (^13^C and ^15^N), were supplemented to the medium at a concentration of 72 mg/L and 90 mg/L, respectively, for the “heavy” medium (Cambridge Isotope Laboratories). For the control medium (non-heavy or “light”), amino acids L-arginine and L-lysine were supplemented at a final concentration of 69 mg/L and 85 mg/L each (Sigma). Both heavy and light medium were supplemented with L-proline at a concentration of 150 mg/L (Sigma). All amino acids were reconstituted in phosphate-buffered saline (PBS) and were filtered through a 0.22-μm filter to obtain a sterile solution (Millipore). Additionally, 10% of dialyzed FBS (Gibco) and AmnioMAX™-C100 Supplement (Gibco) were added to both heavy and light medium, except for the last 48 hours. “Heavy” medium was used to incubate T21 amniocytes, and “light” medium was used to culture CN amniocytes. A minimum of five doubling times was ensured by culturing cells from half a flask of 12 cm^2^-surface area to a flask of 175 cm^2^-surface area at 37°C. Growth media were replaced with fresh media every two to three days over a period of approximately 12 days. When cells become > 90% confluent in a T-175 flask, cells were rinsed with PBS solution three times, and then fresh heavy or light SILAC media were added to the flasks without FBS or AmnioMAX™-C100 Supplement. After 48 hours of incubation, both cells and the supernatant were collected and stored at −20°C until use. Cells were harvested with trypsin and washed with PBS before centrifugation. Cells from preliminary experiments were tested for incorporation of the label after five doubling times.

### Cell lysis protocol for proteomic analysis

Amniotic fluid cell supernatants were lyophilized, preceded by dialysis in 1mM ammonium bicarbonate with two buffer exchanges, using a molecular cutoff of 3.5kDa, for 24h. Amniotic fluid cells were subjected to lysis using cold lysis buffer containing 150 mM NaCl, 20 mM Tris, 6 mM CHAPS, and 1 mM PMSF. Cell pellets were resuspended in 1mM lysis buffer on ice for 10 minutes and sonicated using a probe sonicator for 30 seconds (three times, 10 seconds each). Next, samples were centrifuged at 14000×g for 20 minutes to clear the lysate and only the supernatant portions were retained. The lyophilized supernatant proteins were reconstituted in 50 mM sodium bicarbonate. Coomassie total protein assay (Pierce, USA) was performed to measure total protein amount in all the supernatant and the lysate samples, while each sample was measured in triplicate. Equal amount of “heavy”- and “light”-labelled proteins were combined in 1:1 ratio, and the combined samples were lyophilized to dryness.

### Sample preparation, fractionation, and tandem mass spectrometry

Lyophilized protein samples were reduced in 372 μL of solution, containing 322 μL of 8M urea, 25 μL of water and 25 μL of 200mM DTT at 50°C for 30 minutes. Samples were subjected to acetylation by 500mM iodoacetamide for an hour, and were desalted on a NAP5 column (GE Healthcare). After lyophilization, samples were reconstituted in trypsin solution (1:20, trypsin: protein concentration; 120 μL of 50 mM ammonium bicarbonate; 100 μL methanol; 150 μL dH_2_O) and incubated at 37°C overnight (Promega, sequencing grade modified trypsin).

The detailed description of the sample preparation procedure for 2D-LC-MS/MS can be found in our previous paper [8]. Briefly, the digested peptides, in 120 μL of 0.26 M formic acid in 10% ACN (mobile phase A), were directly loaded onto a PolySULFOETHYL A column. Fractionation was performed using an Agilent 1100 HPLC system for 1 h at a flow rate of 200 uL/min. Ammonium formate (1 M) and 0.26 M formic acid in 10% ACN (mobile phase B) were then used in a linear gradient. The eluent was monitored by UV absorbance at 280 nm. A total of 10 fractions were collected between 20% and 60% of mobile phase B gradient, and were lyophilized to dryness.

Each fraction was resuspended in 80 μL of 95% water, 0.1% formic acid, 5% ACN, 0.02% trifluoroacetic acid (Buffer A) and the digested peptides were purified using OMIX C18 tips (Varian), eluted using 5 μL of 65% acetonitrile solution (0.1% formic acid, 0.02% trifluoacetic acid). Samples were loaded on an Agilent 1100 HPLC by the autosampler onto a 2 cm C18 trap column and the peptides were eluted onto a resolving 5 cm analytical C18 column. The samples were loaded at 15 μL/min for 5 min, then the 103 min gradient was run at 400 nL/min (split from 4 μL/min) starting from 0 to 40% B, followed by 4 min linear gradient to 65%, and finally to 100% B for 1 min. The peptides were subjected to nanoelectrospray ionization followed by tandem mass spectrometry (MS/MS) in an LTQ Orbitrap XL (Thermo, Inc.) coupled online to the HPLC. Data files were created by the Mascot Daemon (version 2.2) and Extract_MSn, and the parameters were: 300 Da minimum mass; 4000 Da maximum mass; automatic precursor charge selection; 10 minimum peaks per MS/MS scan; and 1 minimum scan per group. XCalibur software ver. 2.0.7 (Thermo Fisher Scientific, UK) was used for data acquisition.

### Quantitation of proteins by MaxQuant software

Mass spectra were analyzed using MaxQuant software (version 1.0.0.7), which generates a peak list as well as SILAC- and extracted ion current-based quantitation for SILAC pairs. Raw MS files from all replicates were loaded onto the MaxQuant simultaneously, and identification and quantification of individual peptides were assembled into protein groups. MaxQuant, in conjunction with Mascot (version 2.2, Matrix Science), executes spectral search against a concatenated International Protein Index (IPI) human protein database (version 3.54 containing 39,925 entries) and a decoy database. Parameters included: trypsin enzyme specificity, SILAC double measurements of Lys6 and Arg8, 1 missed cleavage, minimum peptide length of 7 amino acids, minimum of 1 unique peptide, top 6 MS/MS peaks per 100 Da, peptide mass tolerance of 20 ppm for precursor ion and MS/MS tolerance of 0.5 Da. Oxidation of methionine and N-terminal protein acetylation for variable modifications and cysteine caramidomethylation for fixed modification. All entries were filtered using a false positive rate of 1% both at the peptide and protein levels, and false positives were removed. Quantification via normalized H/L ratios was based on minimum of 3 peptide ratio counts. Protein group entries with a normalized ratio significance B score of ≤ 0.05 or significance A score of ≤ 0.05 were retained for further analysis.

### Bioinformatic analysis of amniocyte lysate proteome and candidate selection

The protein reports from MaxQuant were loaded into Microsoft Excel. To visualize and assign functional annotation to over-represented or under-represented proteins, Ingenuity Pathway Analysis (version 8.0, IPA) software was used with IPI numbers as entries, generating a list of canonical pathways that are statistically significant by Fisher’s exact test. A Fisher’s exact test identified canonical pathways most significant to the dataset. Relevant information and annotations for each candidate protein were searched from databases including UniProt, Human Protein Reference Database and Entrez Gene. A protein association network was created where molecules are represented as nodes connected via edges which represent the supporting evidence. Cluster analysis was performed using CIMminer (http://discover.nci.nih.gov/cimminer).

To select candidate proteins that show differential expression due to T21, we applied a series of filters to the list. First, we calculated standard deviation from the control pair (normal to normal ratio) for amniocyte lysate. Applying the values of two standard deviations (2σ; equivalent to 95.45%) from the mean (μ) to the control pair, we created a list of proteins that show significant difference (outside of μ ± 2σ; ~4.55%), and considered these proteins as the “variable proteins”. Next, we applied the same 2σ value to the experimental pairs (T21 amniocytes paired with chromosomally normal amniocytes; pairs 1, 2, and 3), and created separate lists of proteins that show significant difference (outside of μ ± 2σ). We collated these lists together and filtered further by removing the “variable proteins”, reverse hits and known contaminants. Also, we excluded the proteins that fail to show significant p-values (less than 0.05) for either Significance A or Significance B calculated by MaxQuant. “Unknown” or “predicted” proteins were removed. Candidates for verification were selected based on the following additional criteria. First, a protein has to be quantified based on two or more razor peptides and quantification ratios for all peptides should display consistency. Secondly, quantification results with the same pattern of expression should be available for the protein from two experimental pairs. If the result from the third experimental pair is available, it should show similar pattern of expression or not clear differential expression (defined as H/L ratio within the μ ± 0.5σ).

### Sample preparation and SRM method development

For verification, we collected ten additional (five T21 and five CN) amniocyte samples from 15 to 18 weeks of gestation that have been cultured for cytogenetic analysis. Amniocytes were harvested using PBS-based Cell Dissociation Buffer (Gibco) and were gently washed with 1X PBS buffer to remove any external proteins. After centrifugation and aspirating the supernatant, cell pellets were frozen until use. Cell pellets were resuspended with 100 μL of 0.1% RapiGest SF surfactant (Waters) in 25 mM ammonium bicarbonate solution, and were subjected to vortexing and sonication for 3 × 30 s. Total protein for each amniocyte lysate sample was measured by the Bradford assay (Pierce), and the volume was adjusted to extract equal amounts of total protein from individual samples. Lysate proteins (20 μg) were denatured with 0.1% RapiGest SF at 60°C, reduced with 10 mM dithiothreitol, and alkylated with 20 mM iodoacetamide. Samples were then divided into two aliquots (10 μg each) and digested with sequencing grade modified trypsin (Promega) at a trypsin: protein ratio of 1:30, overnight at 37°C. Ninty six femtomoles of heavy ^13^C_6_, ^15^N_2_ L-Lysine-labelled peptide (LSEPAELTDAVK*) of KLK3 protein was added as an internal standard. RapiGest SF was cleaved with 1% trifluoroacetic acid and samples were centrifuged at 1500 x g for 10 min to remove precipitates. Peptides were purified and extracted using 10 μL OMIX C18 tips (Varian), and were eluted using 5 μL of 65% acetonitrile solution with 0.1% formic acid. The final sample was diluted to 130 μL to yield three replicates of 40 μL for injection, so that each sample was analyzed six times.

Peptides were separated on a C18 column-liquid chromatography setup online-coupled to a triple-quadrupole mass spectrometer (TSQ Vantage, Thermo Scientific) using a nanoelectrospray ionization source (nano-ESI, Proxeon A/S). The details of liquid chromatography and MS methods can be found elsewhere [28]. Briefly, a 60-min, three-step gradient was used to load peptides onto the column via an EASY-nLC pump (Proxeon A/S), and peptides were analyzed by an SRM method using the following parameters: predicted CE values, 0.002 m/z scan width, 0.05 s scan time, 0.2 Q1, 0.7 Q3, 1.5 mTorr Q2 pressure and tuned tube lens values.

**Figure 3:**
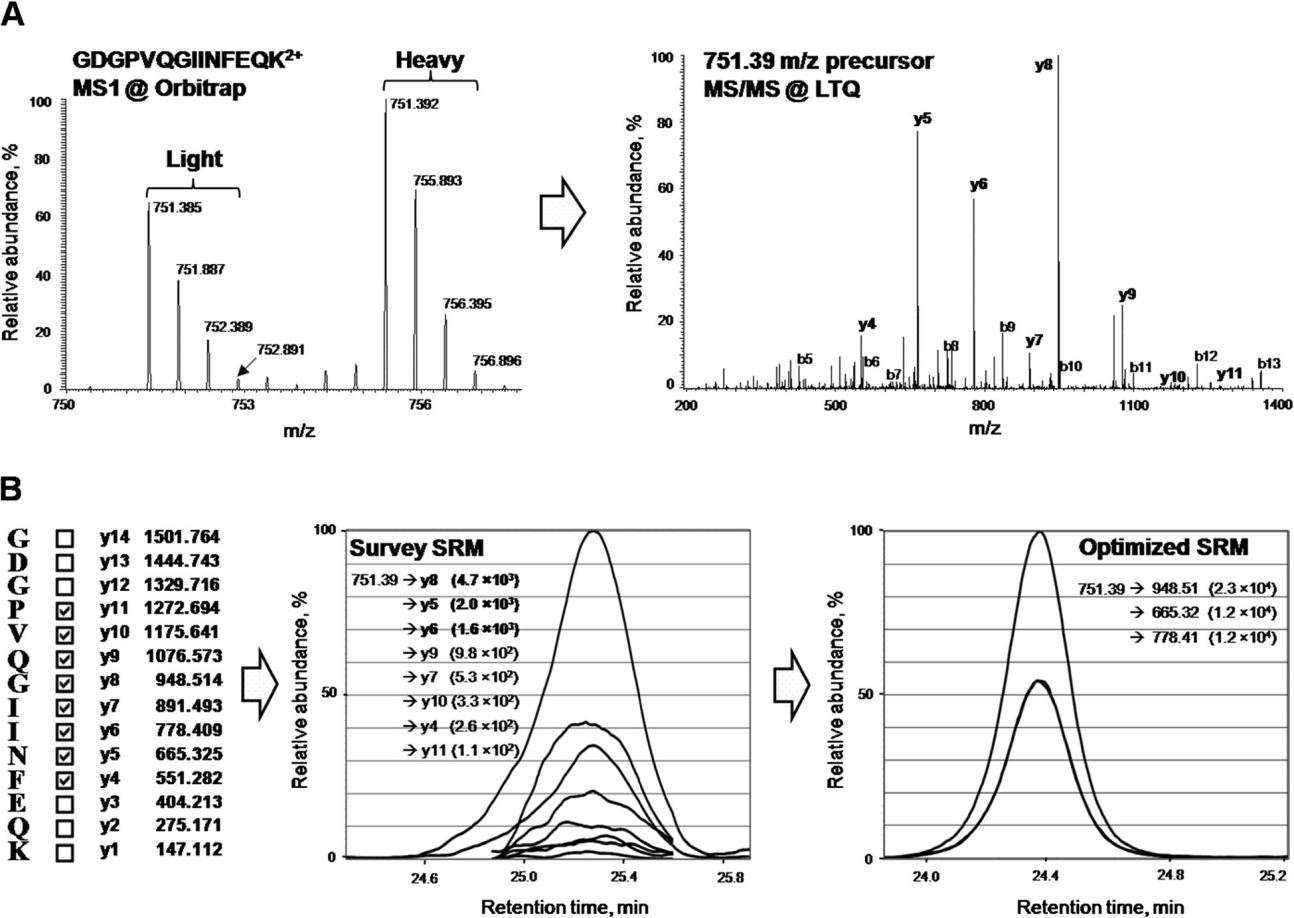
**A schematic representation of SRM method development for candidates and controls.** Differential expression of SOD1 protein and development of SRM assay. **(A)** MS1 spectrum of peptide GDGPVQGIINFEQK in the equimolar mixture of digested lysates of normal (light) and SILAC-labeled Down syndrome (heavy) amniocytes revealed differential expression of SOD1 protein. MS/MS spectrum of peptide GDGPVQGIINFEQK acquired with LTQ-Orbitrap confirmed peptide identity and showed relative intensity of y- and b-fragment ions. **(B)** Survey SRM for peptide GDGPVQGIINFEQK in the triple quadrupole included 8 y-ion transitions, and the three most intense transitions were chosen for the final SRM assay.

SRM method development is depicted in Figure 3. We aimed to identify two unique proteotypic peptides per candidate protein that produce strong peaks with minimal interference. The GPM proteomics database (http://mrm.thegpm.org) was used to select the top 5 peptides per protein based on the intensity of +2 ions. The next step was to confirm their presence from our SILAC proteome results and/or to confirm in SRM atlas (http://srmatlas.org). Peptides of <7 or >20 amino acids in length were eliminated, as well as those with significant +3 ion intensities. Peptides with N-terminal cysteine residues or methionine were avoided. For proteins with multiple peptides that meet the aforementioned criteria, only two peptides with the top intensities were retained. The uniqueness of all peptides were ensured using the Basic Local Alignment Search Tool (BLAST; https://blast.ncbi.nlm.nih.gov/Blast.cgi). Quantification was executed after normalization against a set of ten peptides of high-abundance housekeeping proteins to offset technical variations.

### Analysis of SRM data

Raw files for each sample were analyzed using Pinpoint software (v 1.0) to extract areas under the curve for protein quantification. The statistical software R was used for normalization based on the log_2_-transformed peak areas and subsequent analysis. The first replicate and injection for each sample served as a reference to which the subsequent replicates of the same sample were normalized. A normalization constant was computed by constructing a linear model that was fitted using an M-estimator and robust regression. Normalized values (based on the log_2_-transformed peak areas) for peptide abundance were used to calculate the protein abundance ratio for biological replicates. CVs were computed based on normalized peptide area.

## Abbreviations

CN: Chromosomally normal; CV: Coefficient of variation; DS: Down syndrome; SILAC: Stable isotope labeling with amino acids in cell culture; SRM: Selected reaction monitoring; T21: Trisomy 21.

## Competing interests

The authors declare that they have no competing interests.

## Authors’ contributions

CKJC and EPD participated in the conception and design of the study. CKJC performed cell cultures for SILAC experiments, identified the molecular pathways and analyzed the results. APD developed the SRM methods, carried out the analysis and interpreted the results. GSK performed cell cultures for SRM experiments. CKJC and APD drafted the manuscript. EMM critically revised the manuscript and participated in data analysis and manuscript preparation. SD participated in the optimization of cell cultures for SILAC experiments. AD participated in the statistical analysis and data interpretation. EMM and EPD prepared the final version of the manuscript. All authors read and approved the final manuscript.

## Supplementary Material

Additional file 1 Combined lysate proteome. Click here for file

Additional file 2 Combined supernatant proteome. Click here for file

Additional file 3 Top physiological system development and functions that may be affected by Down syndrome. Click here for file

Additional file 4 Top molecular and cellular functions that may be affected by Down syndrome. Click here for file

Additional file 5 SILAC (H/L) average ratios for seven housekeeping proteins. Click here for file

Additional file 6 Final set of control peptides used for normalization. Click here for file

Additional file 7 Signal intensity for each peptide (raw SRM values). Click here for file

Additional file 8 Normalized areas for each peptide. Click here for file

Additional file 9 Individual and mean CV for each peptide. Click here for file

Additional file 10 SRM ratios for each peptide (DS/CN). Click here for file

Additional file 11 SRM ratios (DS/CN) and mean CV. Click here for file
